# Patterns of health behaviour associated with active travel: a compositional data analysis

**DOI:** 10.1186/s12966-018-0662-8

**Published:** 2018-03-21

**Authors:** Louise Foley, Dorothea Dumuid, Andrew J. Atkin, Timothy Olds, David Ogilvie

**Affiliations:** 1 MRC Epidemiology Unit & UKCRC Centre for Diet and Activity Research (CEDAR), School of Clinical Medicine, University of Cambridge, Box 285 Institute of Metabolic Science, Cambridge Biomedical Campus, Cambridge, CB2 0QQ UK; 2 School of Health Sciences, University of South Australia, GPO Box 2471, Adelaide, South Australia 5001 Australia; 3 School of Health Sciences, Faculty of Medicine and Health Sciences, University of East Anglia, Norwich Research Park, Norwich, NR4 7TJ UK

**Keywords:** Active travel, Walking, Bicycling, Physical activity, Sedentary behaviour, Screen time, Sleep, Compositional data analysis

## Abstract

**Background:**

Active travel (walking or cycling for transport) is associated with favourable health outcomes in adults. However, little is known about the concurrent patterns of health behaviour associated with active travel. We used compositional data analysis to explore differences in how people doing some active travel used their time compared to those doing no active travel, incorporating physical activity, sedentary behaviour and sleep.

**Methods:**

We analysed cross-sectional data from the 2014/15 United Kingdom Harmonised European Time Use Survey. Participants recorded two diary days of activity, and we randomly selected one day from participants aged 16 years or over. Activities were categorised into six mutually exclusive sets, accounting for the entire 24 h: (1) sleep; (2) leisure moderate to vigorous physical activity (MVPA); (3) leisure sedentary screen time; (4) non-discretionary time (work, study, chores and caring duties); (5) travel and (6) other. This mixture of activities was defined as a time-use composition. A binary variable was created indicating whether participants reported any active travel on their selected diary day. We used compositional multivariate analysis of variance (MANOVA) to test whether mean time-use composition differed between individuals reporting some active travel and those reporting no active travel, adjusted for covariates. We then used adjusted linear regression models and bootstrap confidence intervals to identify which of the six activity sets differed between groups.

**Results:**

6143 participants (mean age 48 years; 53% female) provided a valid diary day. There was a statistically significant difference in time-use composition between those reporting some active travel and those reporting no active travel. Those undertaking active travel reported a relatively greater amount of time in leisure MVPA and travel, and a relatively lower amount of time in leisure sedentary screen time and sleep.

**Conclusions:**

Compared to those not undertaking active travel, those who did active travel reported 11 min more in leisure MVPA and 18 min less in screen time per day, and reported lower sleep. From a health perspective, higher MVPA and lower screen time is favourable, but the pattern of sleep is more complex. Overall, active travel was associated with a broadly health-promoting composition of time across multiple behavioural domains, which supports the public health case for active travel.

**Electronic supplementary material:**

The online version of this article (10.1186/s12966-018-0662-8) contains supplementary material, which is available to authorized users.

## Background

Promoting active travel (walking or cycling for transport) has recently gained attention as a public health strategy to enable people and populations to accumulate more daily physical activity [1]. Active travel is cheap or free, accessible to most, and a pragmatic way to embed activity into daily life. Active travel, or its constituent active commuting (walking or cycling to work) have been associated with reduced risk of all-cause mortality [2, 3] and adverse cardiovascular outcomes, [3, 4] a more favourable body composition [5] and greater wellbeing [6] in adults. Conversely, car use has been associated with a less favourable body composition and cardiometabolic profile [7, 8].

Active travel is just one amongst hundreds of activities people undertake across the day. A day may be conceptualised as a ‘time budget’ consisting of 24 h. From this budget, time is allocated to different activities and can be partitioned into different behavioural sets or components, for example, into the proportion of time spent in sleep, sedentary behaviour, light physical activity and moderate to vigorous physical activity (MVPA: which includes active travel [9, 10]). These four activity sets, classified principally on the basis of energy expenditure, together account for all daily time. Low MVPA, [11] high sedentary behaviour, [12, 13] and both short and long sleep durations are associated with unfavourable health outcomes in adults [14, 15].

However they are defined, components of the daily time budget are not independent because increasing time spent in one component necessarily involves reducing time spent in another. Time may be drawn and replaced from different components in myriad different ways, each of which has implications for health [16]. For an example of potential time displacement related to active travel, see Additional file 1. It is clear that, to gain a fuller idea of the likely health outcomes of behaviour change, we need to investigate how individuals restructure their overall time budget to accommodate new behaviours. This necessitates examining the inter-relationship between multiple behaviours, rather than single behaviours in isolation.

Previous research suggests that adding new behaviours is associated with restructuring of time budgets in ways that have both favourable and unfavourable implications for health. A recent randomised controlled trial [17] of a physical activity intervention found that intervention participants significantly increased MVPA, mainly via increased participation in structured exercise and active travel. This time was largely drawn from television watching (sedentary behaviour), but physical activity also displaced sleep. For active travel specifically, preliminary research indicates that increases in active travel are associated with increases in overall MVPA and are not compensated for by reductions in other types of MVPA such as sports [18–20]. However, this finding might not be uniform across all population groups [19], and little is known about the relationship between active travel and other health behaviours such as sleep and sedentary behaviour. A recent systematic review of children’s active travel found sparse and inconsistent evidence of a relationship between active travel and total or screen-based sedentary time [21].

Compositional data analysis is an analytical paradigm that has recently been applied to this field. According to this paradigm, daily time is conceptualised as a mixture of activities or components known as a time-use composition. One recent cross-sectional study indicated relationships between the overall time-use composition and health outcomes in adults [22]. The relative distribution of daily time between sleep, sedentary behaviour, light physical activity and MVPA was significantly associated with body composition, metabolic markers and blood pressure [22]. Of these, MVPA was the most potent health-promoting behaviour, but – critically – this depended on the composition of the rest of the day [22]. Studies in children indicate associations between time-use composition and many outcomes including body composition, [23–25] cardiorespiratory fitness [23, 25], cardiometabolic profile, [25] quality of life [26] and academic performance [27].

The application of a compositional data analysis approach to understanding the patterns of health behaviour associated with specific activities (such as active travel) can inform policy or interventions concerning that activity and a range of others. While compositional data analysis has a long history of application across diverse scientific fields [28], it has been little used in health research. Therefore, the overall objective of the current study was to explore the application of this technique to examine the relative distribution of health behaviour associated with active travel in adults. Here and throughout, we use the term ‘health behaviour’ to refer specifically to physical activity, sedentary behaviour and sleep, acknowledging that this term may incorporate other health behaviours (such as smoking or diet) that were not included in the current study. Specifically, the aims of the current study were to: (a) explore the cross-sectional relationship between active travel and the structure of a 24-h time budget; and (b) identify any differences in this relationship between population sub-groups.

## Methods

### The compositional data analysis paradigm in health research

#### Properties of compositional data

Compositional data are comprised of components which sum to a whole, such as 100%, 1, or in this case 24 h (1440 min). [28] Health researchers may view time use as a composition comprised of sleep and waking behaviours of different metabolic intensities (i.e. sedentary behaviour, light physical activity and MVPA), or as combinations of various mutually exclusive activity domains, such as chores and screen time. Compositions are by nature multivariate, as a composition must comprise at least two components. Compositional information is relative rather than absolute; that is, the information on any individual component is meaningful only by reference to other components. This means that the ratios between components are of primary interest, rather than the absolute values of each component, [29] and the value of the total sum (24 h, one week, one month) is not relevant. For example, in an individual performing one hour of MVPA and 10 h of sedentary behaviour across a 24 h day, the ratio of MVPA to sedentary behaviour is 1:10 or 0.1.

Compositional data exhibit three important properties. Firstly, they are scale invariant, which means that the relative differences between components are maintained regardless of the scale in which they are expressed, such as hours per day [1 h:10 h = 0.1] or percentage of daily time [4.2%:42% = 0.1]) [29]. Secondly, compositional data exhibit sub-compositional coherence, in that the relationship between components is maintained regardless of the presence or absence of other components [29]. In the above example, the ratio of MVPA to sedentary behaviour is still 0.1 regardless of whether sleep (another component of the 24 h time budget) is also reported. Finally, compositions are permutation invariant, as the relative differences between components are the same regardless of the sequence in which components are reported [29].

#### The simplex – A sample space for compositional data

The sample space is defined as the set of all possible values that variables can take. In the example of a coin toss, the sample space consists of heads or tails. Most traditional statistical methods employed in the field of health research (notably regression) assume that data are unconstrained, and therefore operate in real (or Euclidean) space. However, in the case of compositional data, data are constrained to a total sum. Thus, compositional data are represented in a subset of real space known as the simplex, and have a natural geometry, known as Aitchison geometry [30].

The manipulation of variables requires the use of methods congruent to the sample space. For example, the calculation of the arithmetic mean of an unconstrained variable involves adding all observations together and dividing by the number of observations. The arithmetic mean of the numbers 2 and 8 is (2 + 8)/2 = 5. For the same calculation in the simplex, where we are dealing with ratios, perturbation (essentially multiplication) is used in place of addition, and powering (to the power of a negative number) in place of division. As a result, the geometric mean is the most appropriate indicator of central tendency for compositional data, which involves multiplying all observations together and taking the *n*th root. For example, the geometric mean of the numbers 2 and 8 is found by taking the square root of (2 × 8 = 16) = 4. Calculation of the compositional mean involves calculating the geometric mean of each component and adjusting (or ‘closing’) these to the total sum, in this case 24 h [22].

#### Principles of compositional data analysis

The application of traditional statistical methods to compositional data, such as linear regression, can be problematic as these methods are not coherent with the simplex. Even though some pairs of components might appear to be uncorrelated using traditional methods, components are never independent of one another; rather, they are co-dependent to a greater or lesser degree. Thus the inclusion of all components in a model would result in perfect multi-collinearity, negatively biasing the covariance structure of the data [28]. Even the inclusion of more than one component can lead to spurious results.

Compositional data can and should be analysed using methods that account for their properties. A ‘staying in the simplex’ approach can be used, where operations based on Aitchison geometry (e.g. perturbation and powering) are employed. However, the more popular approach is to map compositional data from the simplex into unconstrained real space, where traditional multivariate statistics coherent with real space may be applied. In practice, this is achieved by expressing compositions as log-ratio coordinates. [29] Discussion of the merits of different types of log-ratio coordinate systems may be found elsewhere, [30, 31] but isometric log-ratio (ilr) transformations are most often used. An ilr transformation will produce a set of coordinates numbering one less than the number of components. For example, the four-component composition sleep, sedentary behaviour, light physical activity and MVPA may be expressed as the following set of three normalised log contrasts: (a) sedentary behaviour: sleep; (b) light physical activity: the geometric mean of sleep and sedentary behaviour; and (c) MVPA: the geometric mean of sleep, sedentary behaviour and light physical activity. A positive ilr indicates that the numerator is greater than the denominator for that coordinate, and conversely a negative ilr indicates that the denominator is greater than the numerator. If the ilr is zero, the numerator and the denominator are equal.

Once expressed as ilr coordinates in real space, compositions can be used in statistical models as exposures or outcomes, or both. In the example given above, (c) represents the ratio of MVPA relative to the geometric mean of the remaining components. When used as an exposure, model coefficients for this coordinate correspond to the change in outcome associated with an increase in MVPA relative to compensatory decreases in the remaining components. Alternatively, when used as an outcome, models may be used to predict coordinates based on exposures of interest. In both cases, the ilr coordinates can be back-transformed into proportions, and then into original units (minutes or hours) for interpretation. To date, the small body of literature applying compositional data analysis to health research has used compositions as exposures to explore the aetiology of health or ill health.

#### Zero values in compositional data analysis

Log-ratio coordinates cannot be applied to zero values, meaning that presence of zeros in one or more components prohibits the use of compositional data analysis techniques. In compositional data, zeros can be theorised as ‘rounded’ or ‘essential’. A rounded zero is a small non-zero value that falls below some detection limit, and is thus recorded as zero. For example, the measurement of chemical compositions relies on the sensitivity of the measurement instrument, which may not be able to detect chemicals occurring in very small concentrations. An essential zero is a true zero, indicating the complete absence of that component in the composition. To date, approaches of varying levels of sophistication have been used to impute values in the place of rounded zeros, [32] but the problem of essential zeros remains a core challenge of compositional data analysis. [33] Components containing a large number of zeros or small values are commonly amalgamated with other components. However, this strategy may not be desirable in health research as MVPA typically accounts for a very small proportion of daily time yet is strongly associated with health outcomes.

We now move to describing the current compositional data analysis.

### Study population and design

This study is a secondary analysis of the 2014/15 United Kingdom (UK) Harmonised European Time Use Survey (UKHETUS) [34]. The UKHETUS is a cross-sectional national survey of approximately 7600 UK residents aged eight years or older, conducted between April 2014 and December 2015 [35]. The survey used a multi-stage stratified probability sampling design, generating a random sample of residential addresses using the Postcode Address File and the Land Property Services Agency. The target achieved sample was 5500 households. From a total sample of 11,860 addresses, of which 10,479 were eligible, the response rate was 40.4% or 4238 households [35]. A nominated individual within the household completed a household demographic questionnaire. Following this, all individuals in the household completed an individual demographic questionnaire and two time-use diaries (one on a weekday, one on a weekend day). The study was approved by the Research Ethics Committee of the Department of Sociology (DREC) at the University of Oxford (2014_01_02_R1). For the current analysis we randomly selected one time-use diary from individuals aged 16 years and over.

### Data availability

UKHETUS data are available at 10.5255/UKDA-SN-8128-1.

### Assessment of time-use composition

**Fig. 1:**
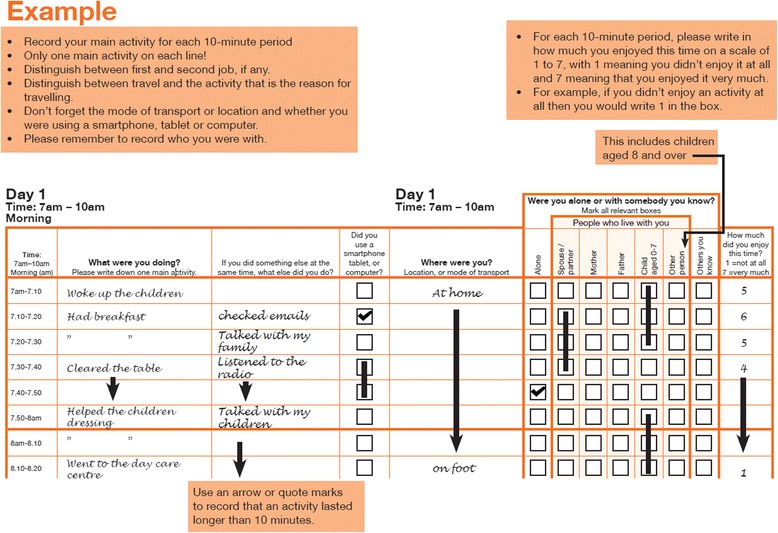
Example time-use diary from the United Kingdom Harmonised European Time Use Survey

Time-use diaries were filled out on the day of interest (Fig. 1). Each diary started at 4 am and covered a full 24 h, in 10-min timeslots. For each timeslot, the participant recorded the primary activity they were undertaking (‘what’ variable) and up to three co-occurring secondary activities. The participant also recorded their location (‘where’ variable) for each timeslot, for example home or work. If they were travelling, the mode of travel was reported under the ‘where’ variable. All responses were given in free text. After the diaries were returned, all free text was coded by an independent rater. For each timeslot, ‘what’ variables were coded into one of 281 a priori individual codes, and ‘where’ variables into one of 38 a priori individual codes [35].

### Quality control

Initial data cleaning was performed in the released dataset, involving the imputation of some missing time according to a set of standard rules [36]. We then applied a series of quality control checks to time-use diaries. Firstly, we conducted a general quality control based on standard procedures used across multiple time-use datasets [37]. We identified diaries with more than 90 min of missing time, which reported less than seven episodes of activity, and were missing two or more of four basic activities (sleeping/resting, eating/drinking, personal care and exercise/travel). We then applied quality control checks specific to our analysis. We identified diaries that did not report a full 24 h of eligible activity codes, where time was coded to one of the following activity codes:

9960 No main activity no idea what it might be.

9970 No main activity some idea what it might be.

9980 Illegible activity.

9990 Unspecified time use.

9991 Not applicable.

9999 Queryable.

We also identified diaries in which no sleep was reported. We removed all diaries failing these quality control checks, on the basis that they were likely incomplete or (in the case of diaries reporting no sleep) atypical representations of the 24 h time budget.

### Definition of exposure (active travel)

We defined active travel as a binary yes/no variable. The participant was categorised as having undertaken some active travel if one of the following codes were reported in the ‘where’ variable in their time-use diary: “travelling on foot” or “travelling by bicycle”. Walking or cycling for recreation was not included in this variable.

### Definition of outcome (time-use composition)

For each participant, we partitioned their time-use diary into six mutually exclusive activity sets (components) according to the primary activity reported in the ‘what’ variable (Additional file 2):

1. Sleep (minutes/day)
2. Leisure MVPA including walking or cycling for recreation (minutes/day)
3. Leisure sedentary screen time (minutes/day)
4. Non-discretionary time comprising work, study, chores and caring duties (minutes/day)
5. Travel including both active and motorised modes (minutes/day)
6. Other including informal help to others and hobbies (minutes/day)

Together, these components accounted for all of the participant’s daily time (24 h or 1440 min). It should be noted that the sleep component represented all sleep occurring between 4 am and 4 am. Thus, it did not necessarily describe an overnight sleep duration, and it incorporated naps undertaken during the day.

We explored patterns of zeros and non-zeros across the defined composition. For the current analysis we treated zero values as rounded, for the following reasons: (a) participants were required to record activity blocks of at least 10 min, meaning that shorter duration activities could have been missed, which is particularly relevant for MVPA; (b) we used only the primary activity to generate the composition, but some relevant activities could have been reported as secondary activities; (c) given the nature of activities included in components (for example “walking and hiking” in the MVPA component), rounding was theoretically possible; and (d) time-use compositions generated from accelerometry (which sample at epochs of 15 s or less) typically have few or no zeros in components [31], which reinforces the suggestion that the cruder level of aggregation in time-use diaries may result in rounded zeros. Therefore, we imputed zero values using a log-ratio data augmentation algorithm, which replaced zeros with small values of less than 10 min, drawing time from the other components. As a sensitivity analysis, we imputed all zero values as one minute.

### Covariates

Covariates hypothesised to confound the association between active travel and time use, which have been used in previous research examining active travel and health behaviours, were selected a priori. Participants reported their age, sex and work status as part of the individual demographic questionnaire. The day of the week of the time-use diary was reported as part of the diary procedure. Age was used as a continuous variable. We used binary variables for sex (male vs. female), work status (working or studying vs. other, including those who answered ‘not applicable’) and day type (weekday vs. weekend).

### Analysis

We used the open source software R (www.r-project.org) and a number of bespoke packages for the analysis of compositional data, including Compositions [38], zCompositions [32] and robCompositions [39].

We explored potential differences between participants included and not included in the analysis, and described the characteristics of the analysis sample. We then conducted an initial descriptive analysis of the raw composition, calculating the arithmetic mean and standard deviation, and the median and interquartile range, of each component. For the imputed composition, we then calculated the geometric mean of each component separately. Finally, we calculated the compositional mean or centre by ‘closing’ the geometric mean of all components to 1440 min. When using the compositional mean, components are adjusted so that they add up to the total. In this case, we used 1440 min or 24 h, a uniform time budget for all participants (i.e. all had the same amount of available time). We examined the variability of the composition using a pair-wise variation matrix, an indicator of dispersion coherent with the simplex, which is broadly equivalent to the standard deviation.

We transformed each participant’s six-component composition into five ilr coordinates for use in regression models. We used the default ilr transformation from the R package Compositions, and the same ilr partitioning system to back-transform the log-ratio coordinates into proportions. The proportions were then adjusted to sum to 1440 for interpretation as minutes per day.

Using the approach described by Martin-Fernández [40], we used compositional multivariate analysis of variance (MANOVA) to contrast the mean time-use composition between individuals reporting some active travel and those reporting no active travel. The null hypothesis was that there was no difference in mean time-use composition between the two groups. A *p* value < 0.05 was taken as evidence to reject the null hypothesis. Models were run in steps, with the first model unadjusted, the second adjusted for age and sex, and the final model adjusted for age, sex, work status and day type.

The MANOVA indicated whether the compositions differed overall between groups, but not which individual components differed. To examine this, it was firstly necessary to estimate adjusted compositional means for each group (i.e. adjusted for age, sex, work status and day type). To estimate the adjusted compositional means, linear regression models were created, with the ilr coordinates as outcome variables and the binary active travel variable as the exposure, along with the other covariates. We used each ilr coordinate as a dependent variable in a unique linear regression model, resulting in five models (one for each coordinate). Using the R package lsmeans [41], we estimated the adjusted mean ilr coordinate value for each of the five ilr coordinates. We did this separately for some and no active travel, resulting in a complete set of five estimated ilr coordinates for each group. Subsequently, we back-transformed these ilr sets to predict model-adjusted six-component compositional means for those reporting some active travel and those reporting no active travel separately.

From this, we adapted the procedure outlined in Martin-Fernández [40] to calculate the log-ratio difference in adjusted compositional means between the two groups. Log-ratio differences are log-transformed ratios where the numerator contains the model-adjusted minutes per day in one component in those reporting some active travel, and the denominator contains the model-adjusted minutes per day in the same component in those reporting no active travel. We then used a bootstrap technique for comparing two populations to construct a 95% bootstrap confidence interval for each separate component. If the confidence interval crossed zero, this indicated that there was no difference between groups with respect to this component [40].

As a final step, we entered interaction terms into the original MANOVA models to explore whether the relationship between active travel and time-use composition differed by sex, work status, age group or day type. If the interaction term was significant (*p* < 0.05), we repeated the adjusted MANOVA models stratifying by that variable (but removing it as a covariate in the model) in order to better elucidate the interpretation of the interaction. We used the same regression model plus bootstrap technique to visualise differences in the individual components in those reporting some active travel and those reporting no active travel, by the stratification variable.

Finally, though the survey used a complex sample design, we did not apply survey weights to the current analysis.

## Results

### Analysis population

We started with 16,533 time-use diaries from 8274 participants. We removed 23 diaries that failed general quality control checks, and 5005 diaries (4988 reporting less than 24 h and 17 reporting no sleep) that failed quality control checks specific to our analysis. Following this, we removed 1182 diaries filled out by those aged under 16 years. Finally, to avoid the issue of clustering by participant, we randomly selected one diary from each participant, leaving us with a final analysis dataset consisting of 6143 diaries from 6143 participants (i.e. one diary from each participant). Compared to those not included, participants included in the analysis were on average older (expected given the age criterion) and significantly more likely to be male and currently working or studying. Characteristics of the analysis sample can be found in Table 1.

**Table 1:** Characteristics of analysis sample (*n* = 6143)

Variable	Mean (SD) or n (%)
Age (years)	47.9 (18.3)
Sex	
male	2905 (47.3)
female	3238 (52.7)
Work status	
working or studying	3949 (64.3)
other	2194 (35.7)
Diary day	
weekday	3093 (50.4)
weekend	3050 (49.7)

*n* number, *SD* standard deviation

### Patterns of active travel

Forty percent (*n* = 2466) of participants reported engaging in active travel on their diary day, and in these individuals the median time spent in active travel was 50 min (interquartile range 60 min). Active travel mostly comprised walking, with 39% (*n* = 2382) of participants reporting any walking, 2% (*n* = 147) reporting any cycling, and 1% (*n* = 63) reporting both.

### Patterns of zeros in the time-use composition

The most common pattern of time-use composition was individuals who reported some of all components apart from leisure MVPA (44%; *n* = 2722). The next most common pattern (20%; *n* = 1253) was individuals who reported some of all components. For leisure MVPA, there were a large number of zero values, with 69% (*n* = 4259) of individuals reporting no time in this component. Because of the quality control procedures, there were no zero values in the sleep component, and the zero values in the other components were more modest – 10% (*n* = 596) for leisure sedentary screen time, 0.1% (*n* = 7) for non-discretionary time, 20% (*n* = 1254) for travel and 10% (*n* = 589) for other.

### Descriptive analysis of the time-use composition

Descriptive characteristics of the raw composition (including zero values) and the imputed composition (in which small numbers were imputed in place of zero values) can be found in Table 2. The different indicators of central tendency provided somewhat different absolute values, particularly between the geometric and compositional means for sleep and non-discretionary time. This is related to the large number of zero values in some components, and is an artefact of the closing procedure used to produce the compositional mean. Coherent with the properties of compositional data described earlier, the relative differences between components are identical when considering either the set of geometric means or the compositional mean.

**Table 2:** Descriptive characteristics of the time-use composition (n = 6143)

Component	Raw composition		Imputed composition	
	Arithmetic mean (SD)	Median (IQR)	Geometric mean	Compositional mean
Sleep (min/day)	499.4 (115.0)	500 (430–570)	482.6	594.4
Leisure MVPA (min/day)	26.8 (55.3)	0 (0–30)	10.5	13.0
Leisure sedentary screen time (min/day)	189.5 (155.0)	160 (70–270)	117.3	144.5
Non-discretionary time (min/day)	482.2 (204.8)	480 (330–640)	423.6	521.8
Travel (min/day)	79.2 (93.7)	60 (20–110)	43.7	53.9
Other (min/day)	162.9 (153.1)	120 (40–240)	91.3	112.5

*IQR* interquartile range, *min* minutes, *MVPA* moderate to vigorous physical activity, *n* number, *SD* standard deviation

The variability of the imputed composition is described in Table 3, which shows variability or proportionality between pairs of components. These can be understood as indicators of the interchangeability of components. In general, the lowest values were found for sleep, indicating low variability of this component, consistent with the fact that sleep is a biological necessity. By contrast, the highest values were found for leisure MVPA, indicating the ability to be interchanged with other components.

**Table 3:** Variation matrix of the time-use composition

	Sleep	Leisure MVPA	Leisure sedentary screen time	Non-discretionary time	Travel	Other
Sleep	0	1.35	0.79	0.34	1.63	1.40
Leisure MVPA	1.35	0	3.60	1.86	3.63	3.75
Leisure sedentary screen time	0.79	3.60	0	1.52	3.34	2.70
Non-discretionary time	0.34	1.86	1.52	0	1.66	2.12
Travel	1.63	3.63	3.34	1.66	0	2.87
Other	1.40	3.75	2.70	2.12	2.87	0

*MVPA* moderate to vigorous physical activity

### Compositional MANOVA

In unadjusted, partially adjusted and fully adjusted models, there was a statistically significant difference in time-use composition between those reporting some active travel and those reporting no active travel (*p* < 0.001). These findings were unchanged in the sensitivity analysis.

### Differences between groups for individual components

**Fig. 2:**
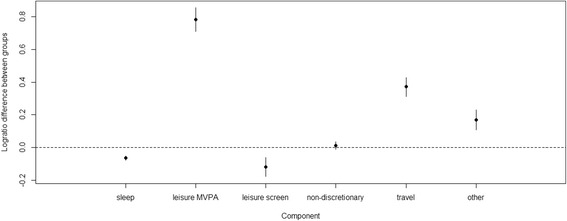
Relative differences in components between those reporting some active travel and those reporting no active travel

The model-adjusted compositional means, presented separately for those reporting some active travel and those reporting no active travel, are displayed in Table 4. Figure 2 shows the log-ratio difference between groups for each component in those reporting some active travel and those reporting no active travel. Values falling above the dotted line indicate that relative time spent in this component was higher in those reporting some active travel compared to those reporting no active travel. Correspondingly, values falling below the line indicate that relative time was lower for that component in those reporting some active travel. Figure 2 indicated a relatively higher amount of time spent in leisure MVPA and travel, and a lower amount of time spent in leisure sedentary screen time and sleep, in those engaging in active travel. The relative differences between groups were greatest for the leisure MVPA component. These findings were unchanged in the sensitivity analysis.

**Table 4:** Model-adjusted^a^ compositional mean by active travel status (n = 6143)

Component	No active travel	Some active travel
Sleep (min/day)^b^	615.7	577.2
Leisure MVPA (min/day)^b^	9.5	20.8
Leisure sedentary screen time (min/day)^b^	163.1	144.7
Non-discretionary time (min/day)	497.0	502.6
Travel (min/day)^b^	42.9	62.2
Other (min/day)^b^	111.9	132.5

*min* minutes

^a^Adjusted for age, sex, work status and day type

^b^Difference between groups statistically significant for this component (*p* < 0.05)

### Effect modification

A statistically significant interaction was found for age, work status and weekday, but not for sex. However, stratified analyses revealed that the overall pattern of time-use composition was not markedly different from the whole sample in any sub-group (Additional file 3).

## Discussion

### Main findings

We found that the structure of a 24-h time budget differed between individuals reporting some active travel and those reporting no active travel. Active travel was associated with relatively greater time spent in leisure MVPA and travel, and relatively lower time in leisure sedentary screen time and sleep. Lower screen time is likely to be favourable from a health perspective, with a difference of 18 min per day between those reporting some active travel and those reporting no active travel. However, interpreting the finding on sleep is more complex. Both short and long sleep durations are associated with poor health in adults, thus the health impacts of lower sleep depend on the baseline level. The pattern of higher leisure MVPA amongst those undertaking active travel may indicate a tendency for people who are physically active in one behavioural domain to also be active in others. The additional 11 min per day of leisure MVPA reported by those undertaking active travel equates to approximately one third of the daily recommended amount of physical activity for adults [42], and was undertaken on top of the MVPA accrued during active travel itself (median 50 min per day). Finally, while statistically significant interactions were found, the overall pattern of behaviour was similar amongst population sub-groups.

### Strengths and limitations

The application of compositional data analysis to health research is a small but rapidly expanding field. To date, this technique has been used to explore the aetiological relationship between time-use composition and health in children [23–27] and adults [22]. To our knowledge, this is the first study in this field using time-use composition as an outcome or dependent variable. Others have suggested this is important to aid intervention development. [43] This is also the first study in adults exploring the association between active travel and multiple health behaviours in tandem.

The strengths of the study include the use of compositional data analysis to account for the co-dependency of behaviours and the inclusion of a large sample of UK adults. In addition, we used time-use diaries to define components at the domain level, providing a finer level of detail than previous compositions defined in terms of energy expenditure using device-based measurement. This allowed us to explore the distribution of particular activities and activity sets, which has been recommended by others as an important avenue for future research [25, 44]. In particular, we explored leisure-time physical activity and sedentary behaviour. Time spent in leisure is likely to be more flexible than time spent for other purposes, and is thus an important consideration for intervention development as it could be drawn upon to accommodate behaviour change.

We also acknowledge the study limitations. In using elements of time use as both exposure and outcome, it might be expected that differences in outcome between groups are a consequence of the re-allocation of active travel time. However, as a proportion of a day active travel time was small (median 50 min per day) whereas the differences we found between groups were larger, suggesting that this is not the only explanation. In addition, this cross-sectional analysis identified potential displacement of time between components, but longitudinal studies exploring individual-level change over time are needed to confirm this. The self-report of activity has known limitations of recall and social biases; however, it is not currently possible to reliably elucidate behavioural domains using objective methods. In our sample, active travel was mostly comprised of walking, but it could be hypothesised that cycling might impose different demands on time budgets (such as showering and changing). Future studies may wish to look at walking and cycling modes separately. Finally, because the primary thrust of this study was methodological, we did not use survey weights in this preliminary analysis, nor did we account for clustering by household. For the current study, the interpretation is limited to this sample and may not be generalisable to the underlying population.

### Comparison with previous work

Our analysis is consistent with previous analyses suggesting that active travel was not associated with reductions in physical activity in other domains [18, 19]. Our findings are also broadly consistent with a previous intervention study indicating that increases in active travel were associated with concurrent reductions in television watching and sleep [17]. This suggests that screen time and sleep may function as ‘time reservoirs’ from which time may be drawn and allocated to other behaviours.

### Implications for research

The principal contribution of this study is to add to an expanding body of work exploring the health case for active travel. However, this study also highlights avenues for further research. Given the limitations of the current study, this analysis could be replicated across other datasets and settings to give a better indication of the generalisability of the findings reported here. In addition, a small number of compositional data analyses to date have simulated the health implications of reallocating fixed durations of time, most commonly 10 min, from one component to another [22, 23, 25]. However, there is a lack of empirical data to confirm whether people do actually reallocate time in the ways and durations modelled. This study and future studies exploring the correlates and determinants of compositions, as well as changes to compositions over time, can begin to provide this type of information. Finally, future studies may wish to model the health implications of a ‘typical’ composition in individuals undertaking active travel.

In the case of time use, the total of the composition does not vary between individuals as everyone has the same amount of time (i.e. 24 h). This means that the importance of relative values is heightened, though the absolute values of components (e.g. MVPA) remain critical in terms of health outcomes. As such, compositional isotemporal analysis is currently being used to explore the health implications of reallocating absolute amounts of time from one component to others [23]. For other types of composition, the total varies between individuals. For example, diet could be expressed as a composition comprised of energy from fat, protein and carbohydrate, which sum to total energy consumed. Here, both the relative composition of macronutrients and the absolute total energy consumed are likely to be important. Methods are currently being developed to incorporate both relative and absolute information together within a compositional data analysis framework [45], and should be applied in future health research.

Finally, this study has implications for the design of future intervention research. Previous studies suggest that some types of policy, infrastructural or behavioural interventions can modify travel behaviour [46]. Future intervention research in this area might usefully examine impacts on time-use composition, in line with calls to explore the wider ripple effects of public health interventions on multiple behaviours [47].

### Implications for policy and practice

Increasing active travel is a stated objective of transport policy in the UK and other countries, as a means to improve health, reduce traffic congestion and reduce greenhouse gas emissions [48]. This study provides evidence to support the public health case for active travel.

This study has further implications for activity guidelines. In recent years, the broadening of the field to consider health behaviours other than physical activity, and the application of compositional data analysis to aetiological research, has promoted a paradigm shift away from recommending increasing or decreasing specific behaviours. Instead, guidance is starting to focus on the optimal composition of time. In Canada, the most recent physical activity guidelines for children and young people are based on a ‘healthy 24 h’ including a set of specific recommendations for sleep, sedentary behaviour, light physical activity and MVPA [49]. This brings with it the need to re-consider population surveillance in order to track progress against these guidelines.

## Conclusions

In conclusion, we found that active travel was associated with a broadly health-promoting composition of time across multiple behavioural domains, which supports the public health case for active travel.

## Additional files

Additional file 1: Consider the example of an individual who takes up active travel by walking part of the journey to work. Firstly, we can consider the direct trade-off of time between transport modes, whereby the individual increases time spent walking (MVPA) and reduces time spent in the car (sedentary behaviour). Replacing sedentary behaviour with MVPA is likely to augment health benefits. However, the indirect or ripple effects on time use are also likely to be important. If the individual who takes up active travel needs to wake up earlier (reduce sleep) to accommodate the new behaviour, then depending on the baseline level of sleep, this may attenuate health benefits. Similarly, if the individual starts walking to work (MVPA) but forgoes a leisurely walk (also MVPA), the displacement of physical activity with physical activity might result in no net health benefit. (DOCX 11 kb)

Additional file 2: **Table S1.** Six-component time-use composition. (DOCX 17 kb)

Additional file 3: **Figure S1.** Relative differences in components between those reporting some or no active travel – participants aged 16–29 years. **Fig. S2** Relative differences in components between those reporting some or no active travel – participants aged 30–59 years. **Figure S3.** Relative differences in components between those reporting some or no active travel – participants aged 60+ years. **Figure. S4.** Relative differences in components between those reporting some or no active travel – participants working or studying. **Figure S5.** Relative differences in components between those reporting some or no active travel – participants not working or studying. **Figure S6.** Relative differences in components between those reporting some or no active travel – weekday. **Figure S7.** Relative differences in components between those reporting some or no active travel – weekend (DOCX 61 kb)

## Abbreviations

ilr: isometric log-ratio
IQR: interquartile range
MANOVA: multivariate analysis of variance
min: minutes
MVPA: moderate to vigorous physical activity
n: number
SD: standard deviation
UK: United Kingdom
UKHETUS: United Kingdom Harmonised European Time Use Survey
