# Supplementary material for: Patterns of health behaviour associated with active travel: a compositional data analysis
Source: Int J Behav Nutr Phys Act. 2018 Mar 21;15:26. doi: 10.1186/s12966-018-0662-8 (PMC5861598; doi:10.1186/s12966-018-0662-8)
Supplement: Supplementary file 1 — Consider the example of an individual who takes up active travel by walking part of the journey to work. Firstly, we can consider the direct trade-off of time between transport modes, whereby the individual increases time spent walking (MVPA) and reduces time spent in the car (sedentary behaviour). Replacing sedentary behaviour with MVPA is likely to augment health benefits. However, the indirect or ripple effects on time use are also likely to be important. If the individual who takes up active travel needs to wake up earlier (reduce sleep) to accommodate the new behaviour, then depending on the baseline level of sleep, this may attenuate health benefits. Similarly, if the individual starts walking to work (MVPA) but forgoes a leisurely walk (also MVPA), the displacement of physical activity with physical activity might result in no net health benefit. (DOCX 11 kb) [file 12966_2018_662_MOESM1_ESM.docx]

Additional file 1

Consider the example of an individual who takes up active travel by walking part of the journey to work. Firstly, we can consider the direct trade-off of time between transport modes, whereby the individual increases time spent walking (MVPA) and reduces time spent in the car (sedentary behaviour). Replacing sedentary behaviour with MVPA is likely to augment health benefits. However, the indirect or ripple effects on time use are also likely to be important. If the individual who takes up active travel needs to wake up earlier (reduce sleep) to accommodate the new behaviour, then depending on the baseline level of sleep, this may attenuate health benefits. Similarly, if the individual starts walking to work (MVPA) but forgoes a leisurely walk (also MVPA), the displacement of physical activity with physical activity might result in no net health benefit.
