# Supplementary material for: Patterns of health behaviour associated with active travel: a compositional data analysis
Source: Int J Behav Nutr Phys Act. 2018 Mar 21;15:26. doi: 10.1186/s12966-018-0662-8 (PMC5861598; doi:10.1186/s12966-018-0662-8)
Supplement: Supplementary file 2 — Table S1. Six-component time-use composition. (DOCX 17 kb) [file 12966_2018_662_MOESM2_ESM.docx]

Additional file 2

Table S1: Six-component time-use composition

| **1. Sleep**  110 Sleep |
| --- |
| **2. Leisure MVPA**  3440 Walking the dog  6000 Unspecified sports and outdoor activities  6100 Unspecified physical exercise  6110 Walking and hiking  6111 Taking a walk or hike that lasts at least 2 miles or 1 hour  6119 Other walk or hike  6120 Jogging and running  6130 Biking skiing and skating  6131 Biking  6132 Skiing or skating  6140 Unspecified ball games  6141 Indoor pairs or doubles games  6142 Indoor team games  6143 Outdoor pairs or doubles games  6144 Outdoor team games  6149 Other specified ball games  6150 Gymnastics  6160 Fitness  6170 Unspecified water sports  6171 Swimming  6179 Other specified water sports  6190 Other specified physical exercise |
| **3. Leisure sedentary screen time**  3713 Shopping for and ordering clothing via the internet  3720 Unspecified household management using the internet  3721 Shopping for and ordering unspecified goods and services via the internet  3722 Shopping for and ordering food via the internet  3724 Shopping for and ordering goods and services related to accommodation via the internet  3725 Shopping for and ordering mass media via the internet  3726 Shopping for and ordering entertainment via the internet  3727 Banking and bill paying via the internet  3729 Other specified household management using the internet  5243 Using internet in the library  5244 Using computers in the library other than internet use  7220 Computing - programming  7230 Unspecified information by computing  7231 Information searching on the internet  7239 Other specified information by computing  7240 Unspecified communication by computer  7241 Communication on the internet  7249 Other specified communication by computing  7250 Unspecified other computing  7251 Skype or other video call  7259 Other specified computing  7330 Computer games  8210 Unspecified tv video or dvd watching  8211 Watching a film on TV  8212 Watching sport on TV  8219 Other specified TV watching  8220 Unspecified video watching  8221 Watching a film on video  8222 Watching sport on video  8229 Other specified video watching |
| **4.Non-discretionary time**  0 Unspecified personal care  210 Eating  300 Other personal care : Unspecified other personal care  310 Other personal care : Wash and dress  390 Other personal care : Other specified personal care  1000 Unspecified employment  1100 Main job : unspecified main job  1110 Main job : Working time in main job  1120 Main job : Coffee and other breaks in main job  1200 Second job : unspecified second job  1210 Second job : Working time in second job  1220 Second job: Coffee and other breaks in second job  1300 Activities related to employment : Unspecified activities related to employment  1310 Activities related to employment : Lunch break  1390 Activities related to employment : Other specified activities related to employment  1391 Activities related to employment : Activities related to job seeking  1399 Activities related to employment : Other specified activities related to employment  2000 Study Unspecified study school or university  2100 Study Unspecified activities related to school or university  2110 Study Classes and lectures  2120 Study Homework  2190 Study Other specified activities related to school or university  2210 Free time study  3000 Unspecified household and family care  3100 Unspecified food management  3110 Food preparation and baking  3130 Dish washing  3140 Preserving  3190 Other specified food management  3200 Unspecified household upkeep  3210 Cleaning dwelling  3220 Cleaning yard  3230 Heating and water  3240 Arranging household goods and materials  3250 Disposal of waste  3290 Other or unspecified household upkeep  3300 Unspecified making and care for textiles  3310 Laundry  3320 Ironing  3330 Handicraft and producing textiles  3390 Other specified making and care for textiles  3410 Gardening  3420 Tending domestic animals  3430 Caring for pets  3490 Other specified gardening and pet care  3500 Unspecified construction and repairs  3510 House construction and renovation  3520 Repairs of dwelling  3530 Making repairing and maintaining equipment  3531 Woodcraft metalcraft sculpture and pottery  3539 Other specified making repairing and maintaining equipment  3540 Vehicle maintenance  3590 Other specified construction and repairs  3600 Unspecified shopping and services  3610 Unspecified shopping  3611 Shopping mainly for food  3612 Shopping mainly for clothing  3613 Shopping mainly related to accommodation  3614 Shopping or browsing at car boot sales or antique fairs  3615 Window shopping or other shopping as leisure  3619 Other specified shopping  3620 Commercial and administrative services  3630 Personal services  3690 Other specified shopping and services  3710 Household management not using the internet  3800 Unspecified childcare  3810 Unspecified physical care & supervision of a child  3811 Feeding the child  3819 Other and unspecified physical care & supervision of a child  3820 Teaching the child  3830 Reading playing and talking with child  3840 Accompanying child  3890 Other or unspecified childcare  3910 Unspecified help to a non-dependent eg injured adult household member  3911 Physical care of a non-dependent eg injured adult household member  3914 Accompanying a non-dependent adult household member eg to hospital  3919 Other specified help to a non-dependent adult household member  3920 Unspecified help to a dependent adult household member  3921 Physical care of a dependent adult household member eg Alzheimic parent  3924 Accompanying a dependent adult household member eg Alzheimic  3929 Other specified help to a dependent adult household member |
| **5. Travel**  9000 Travel related to unspecified time use  9010 Travel related to personal business  9100 Travel to/from work  9110 Travel in the course of work  9120 Travel to work from home and back only  9130 Travel to work from a place other than home  9210 Travel related to education  9230 Travel escorting to/ from education  9310 Travel related to household care  9360 Travel related to shopping  9370 Travel related to services  9380 Travel escorting a child other than education  9390 Travel escorting an adult other than education  9400 Travel related to organisational work  9410 Travel related to voluntary work and meetings  9420 Travel related to informal help to other households  9430 Travel related to religious activities  9440 Travel related to participatory activities other than religious activities  9500 Travel to visit friends/ relatives in their homes not respondents household  9510 Travel related to other social activities  9520 Travel related to entertainment and culture  9600 Travel related to other leisure  9610 Travel related to physical exercise  9620 Travel related to hunting & fishing  9630 Travel related to productive exercise other than hunting & fishing  9710 Travel related to gambling  9720 Travel related to hobbies other than gambling  9800 Travel related to changing locality  9810 Travel to holiday base  9820 Travel for day trip/ just walk  9890 Other specified travel |
| **6. Other**  [All other activity codes] |
