# Supplementary material for: Patterns of health behaviour associated with active travel: a compositional data analysis
Source: Int J Behav Nutr Phys Act. 2018 Mar 21;15:26. doi: 10.1186/s12966-018-0662-8 (PMC5861598; doi:10.1186/s12966-018-0662-8)
Supplement: Supplementary file 3 — Figure S1. Relative differences in components between those reporting some or no active travel – participants aged 16–29 years. Fig. S2 Relative differences in components between those reporting some or no active travel – participants aged 30–59 years. Figure S3. Relative differences in components between those reporting some or no active travel – participants aged 60+ years. Figure. S4. Relative differences in components between those reporting some or no active travel – participants working or studying. Figure S5. Relative differences in components between those reporting some or no active travel – participants not working or studying. Figure S6. Relative differences in components between those reporting some or no active travel – weekday. Figure S7. Relative differences in components between those reporting some or no active travel – weekend (DOCX 61 kb) [file 12966_2018_662_MOESM3_ESM.docx]

Additional file 3


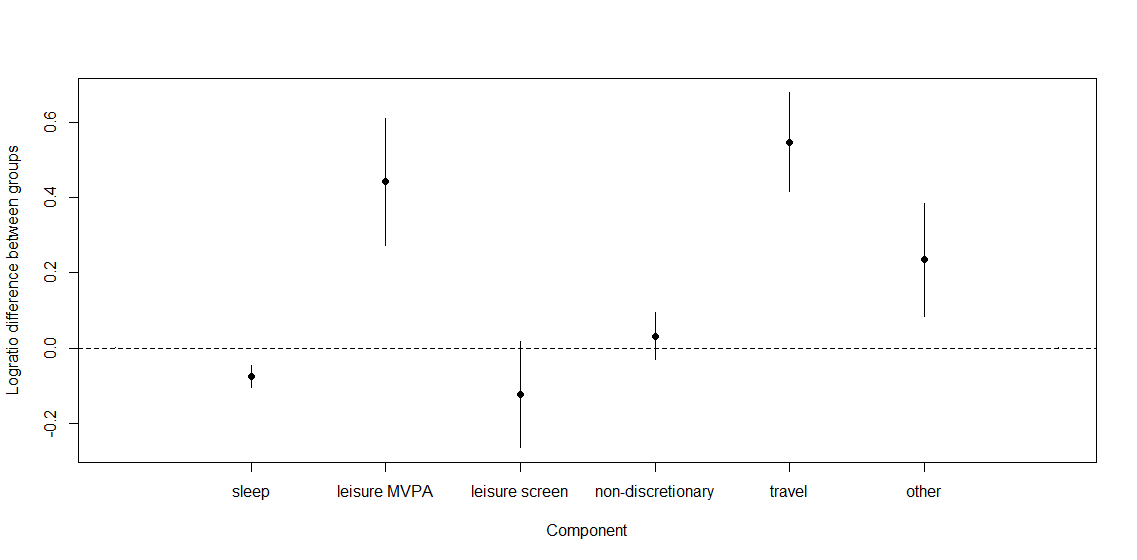


**Figure S1**: Relative differences in components between those reporting some or no active travel – participants aged 16-29 years


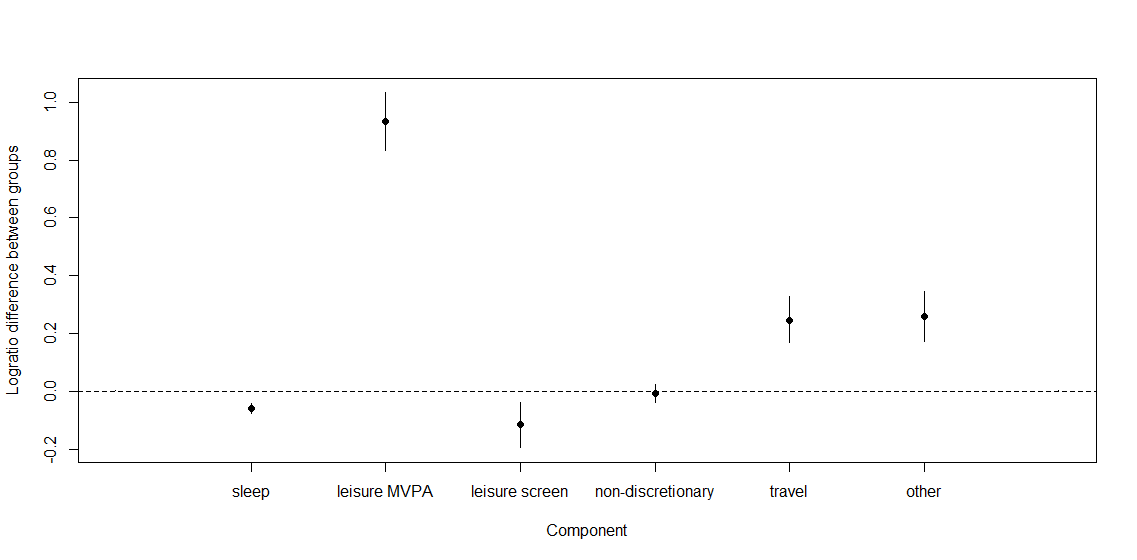


**Figure S2**: Relative differences in components between those reporting some or no active travel – participants aged 30-59 years


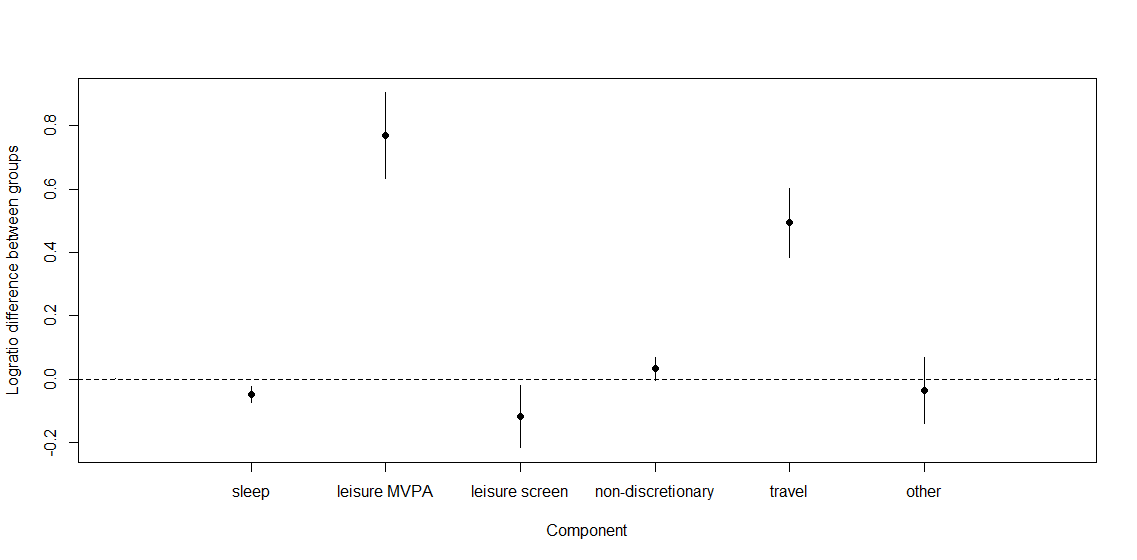


**Figure S3**: Relative differences in components between those reporting some or no active travel – participants aged 60+ years


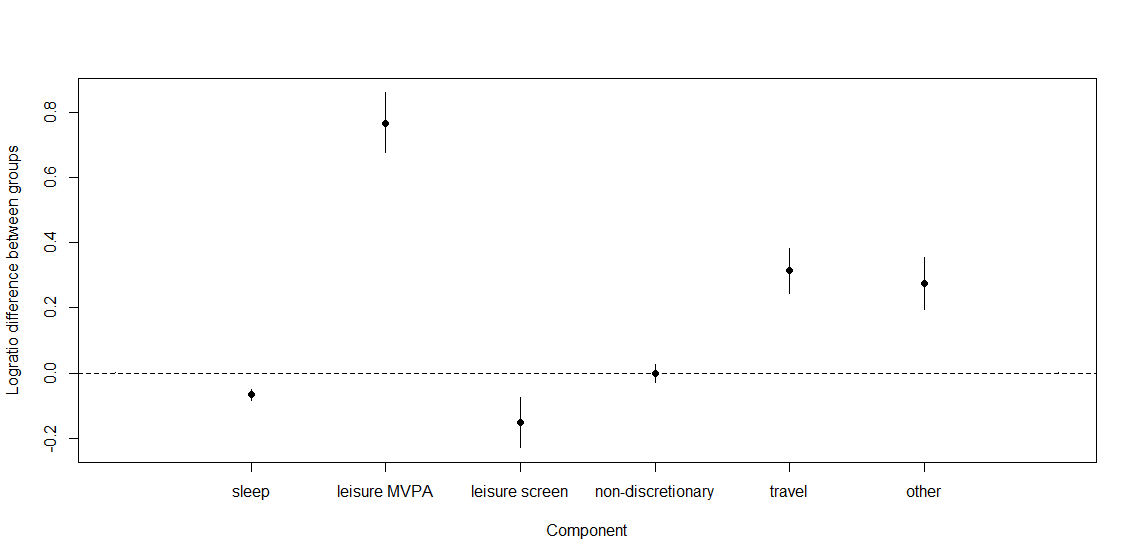


**Figure S4**: Relative differences in components between those reporting some or no active travel – participants working or studying


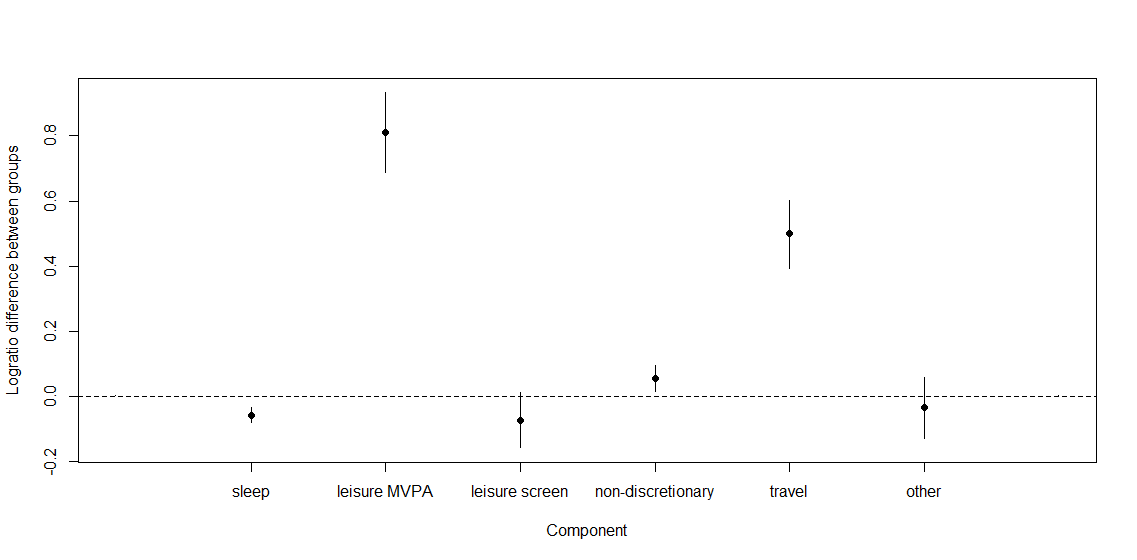


**Figure S5**: Relative differences in components between those reporting some or no active travel – participants not working or studying


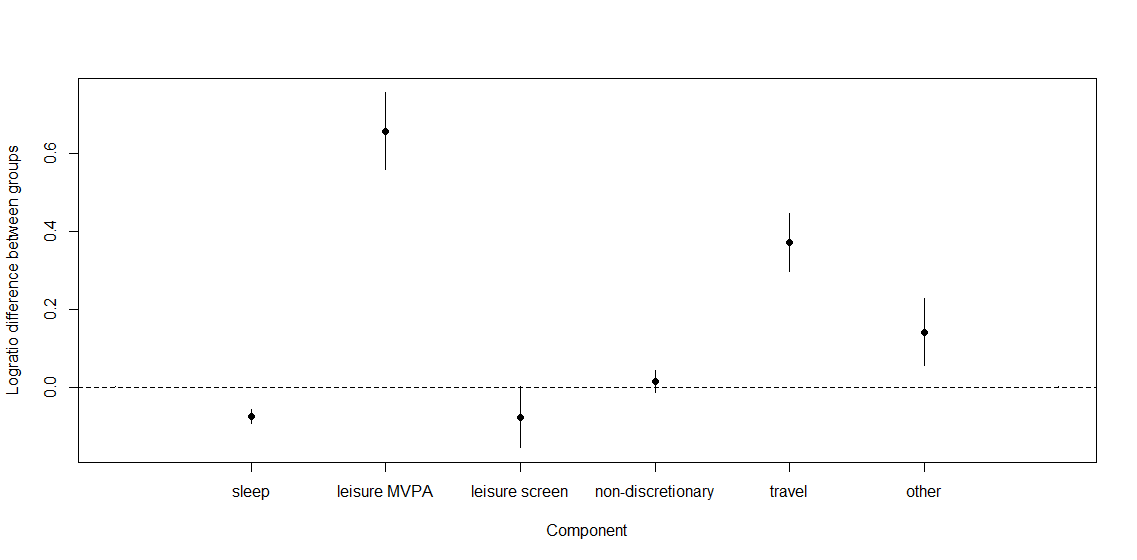


**Figure S6**: Relative differences in components between those reporting some or no active travel – weekday


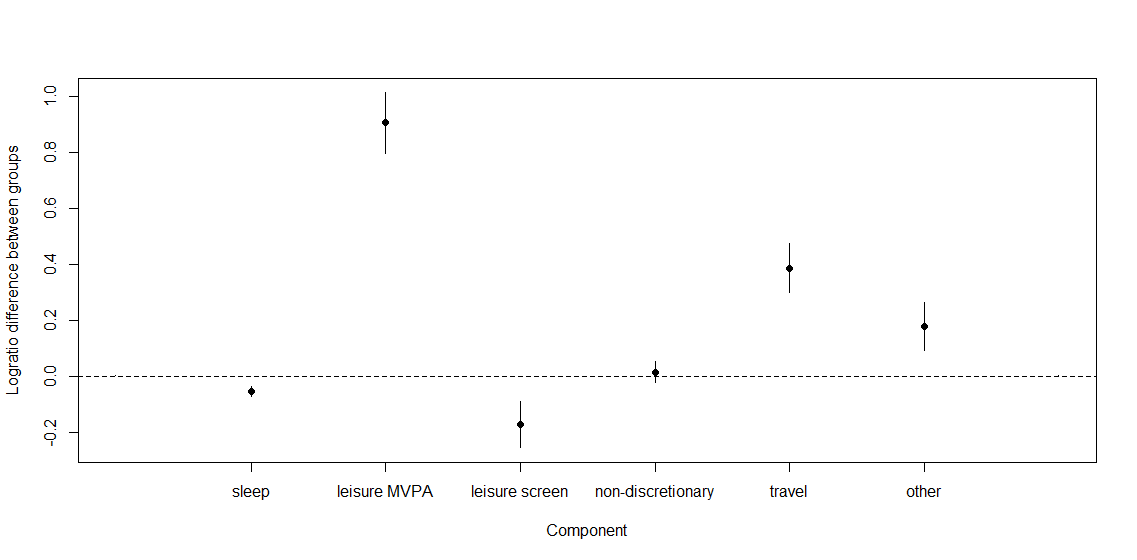


**Figure S7**: Relative differences in components between those reporting some or no active travel – weekend
